# Unexpected pulmonary tumour in a young woman

**DOI:** 10.1136/jclinpath-2018-205259

**Published:** 2019-07-26

**Authors:** Geoffroy Boulle, Margot Dupeux, Marie-Christine Charpentier, Fréderique Larousserie, Jean François Régnard, Diane Damotte, Audrey Mansuet-Lupo

**Affiliations:** 1 Department of Pathology, HUPC, Cochin Hospital, Paris, France; 2 INSERM UMR 1138, Team 'Cancer, Immune Control, and Escape' Cordeliers Research Center, Paris, France; 3 Department of Thoracic Surgery, HUPC Cochin Hospital, Paris, France

**Keywords:** lung cancer, sarcomas, surgery

## Clinical question

A 21-year-old woman presented with an upper left lobe mass, discovered in a context of asthenia, dyspnoea, wheezing, flushes and evening fever. Initial CT imaging revealed a heterogeneous mass predominantly endobronchial into the bronchus of the lingula with latero-aortic and perihilar adenopathies. Positron emission tomography-CT scan found a hypermetabolism of the tumour (maximum standard uptake value (SUVmax=10) as well as in mediastinal lymph nodes (SUV=2.2). Initial fibroscopy performed found a stenosing endoluminal tumour. Left superior lobectomy and mediastinal lymph node dissection were performed.

Review the high quality, interactive digital Aperio slide at http://virtualacp.com/JCPCases/jclinpath-2018-205259/ and consider your diagnosis.

## Q1: What is your diagnosis?

Hematoxylin eosin safron (HES) slide 1:

- Carcinoïd tumour
- Pulmonary myxoid sarcoma
- Inflammatory myofibroblastic tumour
- Myxoid liposarcoma
- Pulmonary haematoma

## Q2: What additional analyse(s) do you perform?

- *EWSR1* fluorescence in situ hybridisation *(*FISH)
- *ALK* immunohistochemistry
- Synaptophysin immunohistochemistry
- Molecular analysis for lung carcinoma (*EGFR*, *ALK*, *ROS1*)
- Nothing else

­

The correct answers are after the discussion.

## Discussion

Pulmonary myxoid sarcoma (PMS) is a very rare low-grade tumour, with only about 15 cases described in the literature.^1 2^ According to available data, this disease has not a male or female predominance, with an age range of 28–68 years. The clinical presentation is usually various; the symptoms may be cough, chest pain, haemoptysis or asymptomatic and could be accidentally discovered. Here, the macroscopic examination of the left superior lobe found a proximal tumour of 7×6×4 cm, well defined, growing in the upper lobar bronchus, following the bronchial tree with minimal areas of pulmonary infiltration. Microscopically tumorous cells appeared monomorphic, fusiform, not atypical and dispersed in a myxoid substance. There were very few mitoses and no necrosis. The lymph node contained a follicular lymphoid hyperplasia without tumour localisation. Tumorous cells were negative for AE1/AE3 (Dako), actin (1A4, Dako), desmin (D33, Dako) and CD34 (Qbend-10, Dako) but 80% of tumour cells were positive for epithelial membrane antigen (E29, Dako). A FISH was performed on formalin-fixed paraffin-embedded tumour tissues and found a translocation of *EWSR1*, which was confirmed by the next-generation sequencing (NextSeq 550 System, illumina) and correspond to the fusion transcript *EWSR1*/*ATF1*. The genetic characteristic of PMS is often the (2; 22) (q33; q12) translocation with the *EWSR1-CREB1* fusion gene. *EWSR1* is found in most chromosomal translocations of sarcomas, with nearly 16 types of partners indexed.^3^ *EWRS1/ATF1* can be found rarely in this type of tumour. *ATF1* encodes for a cyclic AMP protein responsive element which is constitutively product after the translocation with *EWRS1.* 4 This fusion with the partenaire *ATF1*, however, is not specific for myxoid sarcoma, since it is also found in clear cell sarcoma and angiomatoid fibrous histiocytoma.^2–5^ The differential diagnosis is mainly pulmonary mesenchymal chondrosarcoma and other myxoid tumours, as myxoid liposarcoma.^6 7^

The immediate operative follow-up was simple, and the patient received no further treatment. At 6 months of surgery, the patient had no evidence of clinicoradiological recurrence. The risk of relapse is low; however, there is a metastatic potential with few described cases of secondary cerebral, renal and pulmonary localisations.

## Answers

Q1 Pulmonary myxoid sarcoma; Q2 *EWSR1* fluorescence in situ hybridisation *(*FISH).

**Take home messages**

- Mesenchymal tumours of the lung are very rare; the most frequent is pulmonary hamartoma, a benign tumour.
- Pulmonary myxoid sarcoma (PMS) is a low-grade tumour, often localised partially endobronchial and exceptionally metastatic.
- The diagnosis of PMS is morphological and confirmed by the presence of *EWSR1* translocation.
